# Comparing usage of a web and app stress management intervention: An observational study

**DOI:** 10.1016/j.invent.2018.03.006

**Published:** 2018-04-21

**Authors:** Leanne G. Morrison, Adam W.A. Geraghty, Scott Lloyd, Natalie Goodman, Danius T. Michaelides, Charlie Hargood, Mark Weal, Lucy Yardley

**Affiliations:** a Department of Psychology, Faculty of Social, Human, and Mathematical Sciences, University of Southampton, Southampton, Hampshire, UK; b Primary Care and Population Sciences, Faculty of Medicine, University of Southampton, Southampton, Hampshire, UK; c Redcar & Cleveland Borough Council, Redcar, Yorkshire, UK; d Health and Social Care Institute, School of Health and Social Care, Teesside University, Middlesbrough, Tees Valley, UK; e Fuse, Centre for Translational Research in Public Health, Newcastle University, Newcastle upon Tyne, Tyne and Wear, UK; f Centre for Public Policy and Health, School of Medicine, Pharmacy and Health, Durham University, Stockton on Tees, UK; g Gateshead Council, Gateshead, Tyne and Wear, UK; h Electronics and Computer Science, University of Southampton, Southampton, Hampshire, UK; i Nuffield Department of Primary Care Health Sciences, Medical Sciences Division, University of Oxford, Oxford, UK

**Keywords:** Internet, Mobile applications, Data analysis, Health, Behavioural research, Usage

## Abstract

Choices in the design and delivery of digital health behaviour interventions may have a direct influence on subsequent usage and engagement. Few studies have been able to make direct, detailed comparisons of differences in usage between interventions that are delivered via web or app. This study compared the usage of two versions of a digital stress management intervention, one delivered via a website (Healthy Paths) and the other delivered via an app (Healthy Mind). Design modifications were introduced within Healthy Mind to take account of reported differences in how individuals engage with websites compared to apps and mobile phones. Data were collected as part of an observational study nested within a broader exploratory trial of Healthy Mind. Objective usage of Healthy Paths and Healthy Mind were automatically recorded, including frequency and duration of logins, access to specific components within the intervention and order of page/screen visits. Usage was compared for a two week period following initial registration. In total, 381 participants completed the registration process for Healthy Paths (web) and 162 participants completed the registration process for Healthy Mind (app). App users logged in twice as often (*Mdn* = 2.00) as web users (*Mdn* = 1.00), *U* = 13,059.50, *p* ≤ 0.001, but spent half as much time (*Mdn* = 5.23 min) on the intervention compared to web users (*Mdn* = 10.52 min), *U* = 19,740.00, *p* ≤ 0.001. Visual exploration of usage patterns over time revealed that a significantly higher proportion of app users (*n* = 126, 82.35%) accessed both types of support available within the intervention (i.e. awareness and change-focused tools) compared to web users (*n* = 92, 40.17%), *χ*^2^(1, *n* = 382) = 66.60, *p* < 0.001. This study suggests that the digital platform used to deliver an intervention (i.e. web versus app) and specific design choices (e.g. navigation, length and volume of content) may be associated with differences in how the intervention content is used. Broad summative usage data (e.g. total time spent on the intervention) may mask important differences in how an intervention is used by different user groups if it is not complemented by more fine-grained analyses of usage patterns over time. Trial registration number: ISRCTN67177737.

## Highlights

• Modifying the design and delivery of theoretical intervention content may alter how that content is used and received.
• App users logged in twice as often but spent half as much time on the intervention compared to web users.
• App users accessed more of the available intervention tools compared to web users.
• Fine-grained individual-level data can enable comparison of how an intervention is used over time by different user groups.

## 1 Introduction

Health and behaviour change interventions delivered using digital technology offer the potential to automatically collect rich data on how the intervention has been used by individual participants. This data can range from summative metrics (e.g. number of logins, duration of logins, frequency of visits to particular intervention components) to fine-grained individual-level data detailing each individual's flow through the intervention (e.g. what has been visited, for how long and in what order) (Morrison and Doherty, 2014). Analysis of this data is crucial for identifying factors associated with variations in intervention usage (e.g. design factors, user characteristics), and for informing understanding of the relationship between intervention usage and health-related outcomes.

To date, numerous intervention evaluation studies have used summative metrics to report broad patterns of intervention usage and how these relate to outcomes (e.g. Glasgow et al., 2011; Richardson et al., 2013; Whitton et al., 2015). Other work has sought to analyse user characteristics associated with greater usage of or exposure to the intervention content (e.g. Brouwer et al., 2009; Van't Riet et al., 2010) or compare usage across different interventions that are focused on a particular health condition or behaviour (Nelson et al., 2016). Such analyses can inform conceptual models of engagement that identify user or design-related factors that may enhance engagement with an intervention platform and for identifying user groups for whom the intervention is likely to be most engaging and effective (Perski et al., 2016).

In addition to broad summative-level metrics, some digitally delivered interventions offer the opportunity to collect and analyse rich individual-level data on how the intervention is used and engaged with over time. Systematic analysis and interpretation of such data is methodologically challenging (Morrison and Doherty, 2014) and there is a lack of guidance available in how best to approach usage analyses to enable comparability and applicability across studies. Visual exploration of intervention usage has shown promise as a way to supplement summative usage metrics by providing a more efficient means of exploring large, richer data sets at finer levels of granularity (e.g. Arden-Close et al., 2015; Morrison and Doherty, 2014).

Increasing proliferation of digital interventions and rapid advancement in technology also raises empirical questions about the choice of platform for delivering health behaviour change interventions. For example, to what extent are usage patterns influenced by the design of interventions delivered through different digital platforms (i.e. web versus app)? Few studies have directly compared the usage of interventions delivered through different digital platforms. Morrison et al. (2014) compared usage of an online weight management intervention when provided with or without a supplementary app. This study suggested that combining web and app delivery can help to improve users' awareness of their personal weight management goals, but did not directly compare web and app delivery of the intervention content. Quinonez et al. (2016) directly compared email versus SMS delivery of an intervention to promote physical activity. Their analysis demonstrated that email delivery was associated with lower rates of drop out and higher self-reported engagement with the tailored physical activity messages (e.g. number of messages received and read). This study suggests that there may be differences in how interventions are used and responded to as a result of how they are delivered through the digital technology.

Comparing web and app delivery of an identical intervention is problematic as qualitative research suggests that individuals are likely to engage differently with websites and apps in their day-to-day lives. Dennison et al. (2013) highlighted that apps were perceived as disposable and not necessarily seen as a long-term commitment. Morrison et al. (2014) also found that app content was typically used on-the-go, sporadically for shorter periods of time than web content. Mobile screen space is also more limited than on PCs. Thus, comparison of exactly the same content delivered via different digital platforms (as reported in Quinonez et al., 2016) is likely to influence the conclusions drawn about usage and engagement as no account is made in the design and delivery of the intervention of how individuals use different digital platforms within their day-to-day lives. Duplicating a design originally intended to be accessed via email or on a PC may well result in lower engagement when accessed through mobile platforms if appropriate modifications for mobile delivery are not made (Lattie et al., 2016).

To our knowledge, this study is one of the first to provide a detailed, direct comparison of usage of a web and app intervention that made modifications to take account of how these different platforms are used within individuals' daily lives. The aims of the study were to:

1. Compare patterns of usage between a web and app stress management intervention.
2. Compare insights gained from two approaches to analysing intervention usage data. These included descriptive statistics of summative level data versus visual exploration of individual-level data and temporal usage of the intervention.

The design differences between the web and app versions mean that users did not receive identical versions of the intervention. This study therefore compares two intervention packages that share the same underlying ‘theoretical action components’ (i.e. to support users in applying mindfulness-based and cognitive behavioural strategies to help manage stress and improve mental wellbeing), but differ in their ‘instantiation’ (i.e. sequence of delivery, volume of content) (Mohr et al., 2015). The aim of the presented analysis is not then to draw conclusions about whether web or app delivery of identical intervention content is associated with more desirable usage patterns, but rather to provide insight about how choices in the delivery of intervention content may relate to potentially crucial differences in usage and receipt of the intervention.

## 2 The interventions

### 2.1 Healthy Paths (web)

Healthy Paths through Stress (short name ‘Healthy Paths’) is an online intervention that offers a range of evidence-based tools for managing emotional distress. Healthy Paths was created using LifeGuide intervention authoring software (http://www.lifeguideonline.org) following a person-based approach (Geraghty et al., 2016). The tools provided by Healthy Paths are drawn from mindfulness-based approaches and cognitive behavioural therapy (see Table 1). Each tool was designed to support participants to improve awareness of their thoughts or behaviours or support change in thinking patterns and behaviours. The content and design of Healthy Paths was developed by a multi-disciplinary team comprised of psychologists and clinicians in close collaboration with primary care patients who were experiencing distress primarily stemming from stressful life circumstances. Healthy Paths was designed to support users in managing emotional distress and was not intended as an intervention for psychological disorders (e.g. depressive disorder or generalised anxiety disorder).

**Table 1:** Tools included within Healthy Paths (web) and Healthy Mind (app).

Healthy Paths	Healthy Mind	Description
Walking with awareness (awareness)	*✓	Guided walking activity to encourage greater conscious awareness of the experience of walking (e.g. bodily sensations, surrounding environment).
Monitoring thoughts (awareness)	My daily reactions (awareness)	Identify and record physical, behavioural, and affective reactions to daily stressful events.
Monitoring reactions (Awareness)		
3 minute breathing space (awareness)	⁎✓	Guided 3-minute breathing exercise.
Body scan (awareness)	✓	Guided 10-minute body scan exercise.
Connect with others (change)	*✓	Create/select from plans to spend time with other people.
Monitoring pleasant activities (awareness)	Enjoyable moments (change)	Record and reflect on how often one engages in pleasant activities (e.g. reading, taking a long bath, gardening).
Increasing pleasant activities (change)		
Sleep well (change)	✓	Select and review goals for improving sleep quality.
Self-kindness	*✓	Guided exercises to cultivate self-compassion.
Positive thought starting	✓ (Renamed ‘positive thinking’)	Create/select from a list of positive thoughts (e.g. I always learn something new from dealing with a stressful situation).

* Starter tool within the Healthy Mind app.

✓ Tool remained unchanged in app.

### 2.2 Healthy Mind (app)

Healthy Mind is an Android app that was adapted from the Healthy Paths website and was created using the Life Guide Toolbox software (Hargood et al., 2014). Healthy Mind provides the same basic content as Healthy Paths, that is, the same range of ‘tools’ (see Table 1). However, the volume and delivery of content provided by the app was adapted in specific ways to better accommodate how individuals were perceived to routinely engage with their mobile phones on a day-to-day basis. Key differences between the web and app versions of the intervention are described in detail in Section 2.3.

### 2.3 Summary of key differences

Three design changes were introduced in the app version of the intervention: 1) simplifying the navigation of the app and introducing a tool unlocking feature (see Section 2.3.1), 2) simplifying and reducing the content of the app to enable faster access to the core tools (see Section 2.3.2), and 3) use of push notifications to suggest specific tools (see Section 2.3.3). These design changes were intended to encourage repeated engagement given prior qualitative research suggesting that usage of apps can be perceived as a short-term commitment (Dennison et al., 2013).

#### 2.3.1 Navigation

**Fig. 1:**
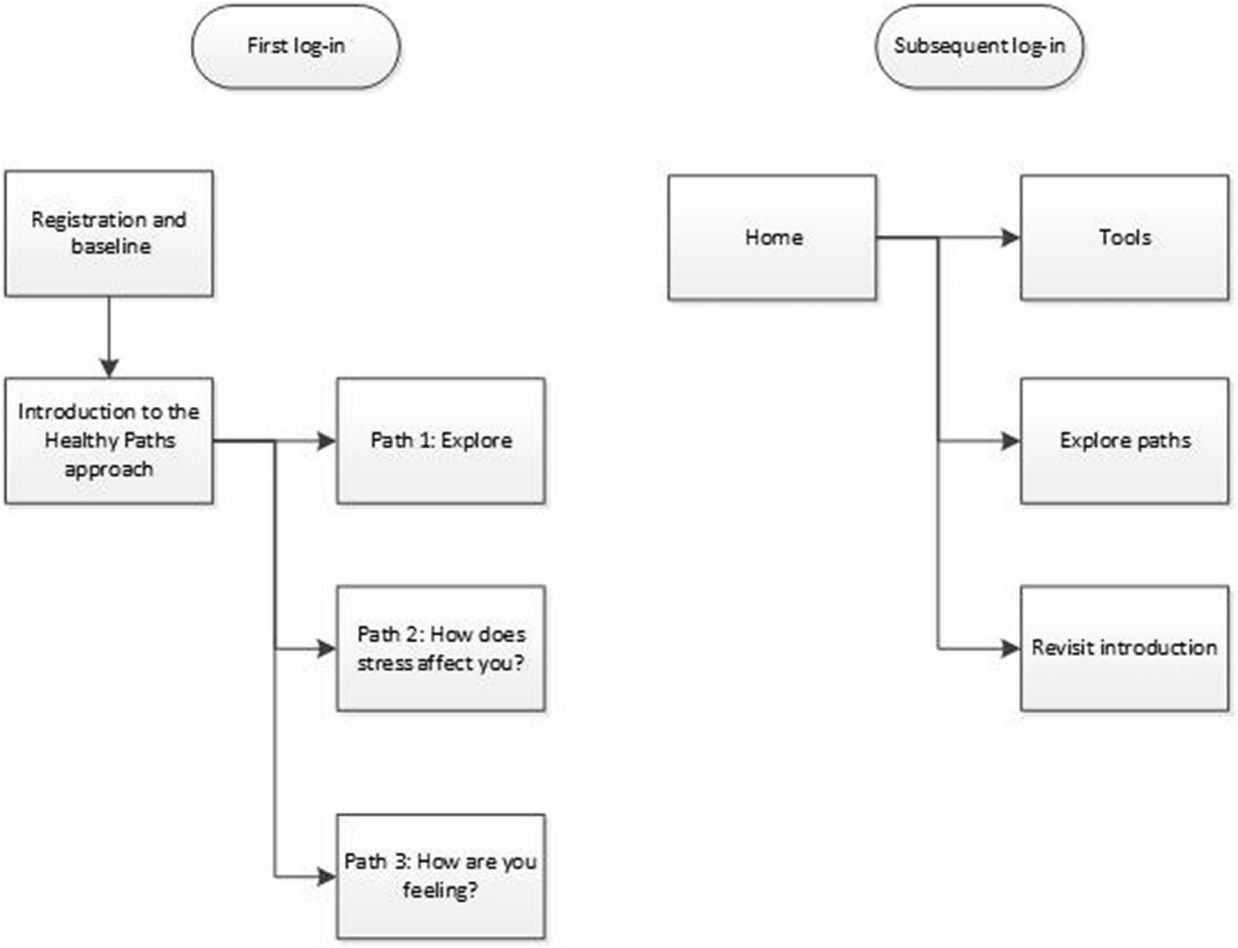
Navigation structure for Healthy Paths (web).

On first access to the Healthy Paths website users were guided through a series of introductory pages that described the aims, contents and benefits of using Healthy Paths (see Fig. 1). Following this introduction, users were invited to explore the tools within the intervention in one of three different ways: 1) free exploration of the tools, 2) tailored tool suggestions based on personal experiences of stress, 3) tailored tool suggestions based on current emotional state. On subsequent log-ins, users were free to access the tools through any of these three pathways. All tools within the website were available from the outset.

**Fig. 2:**
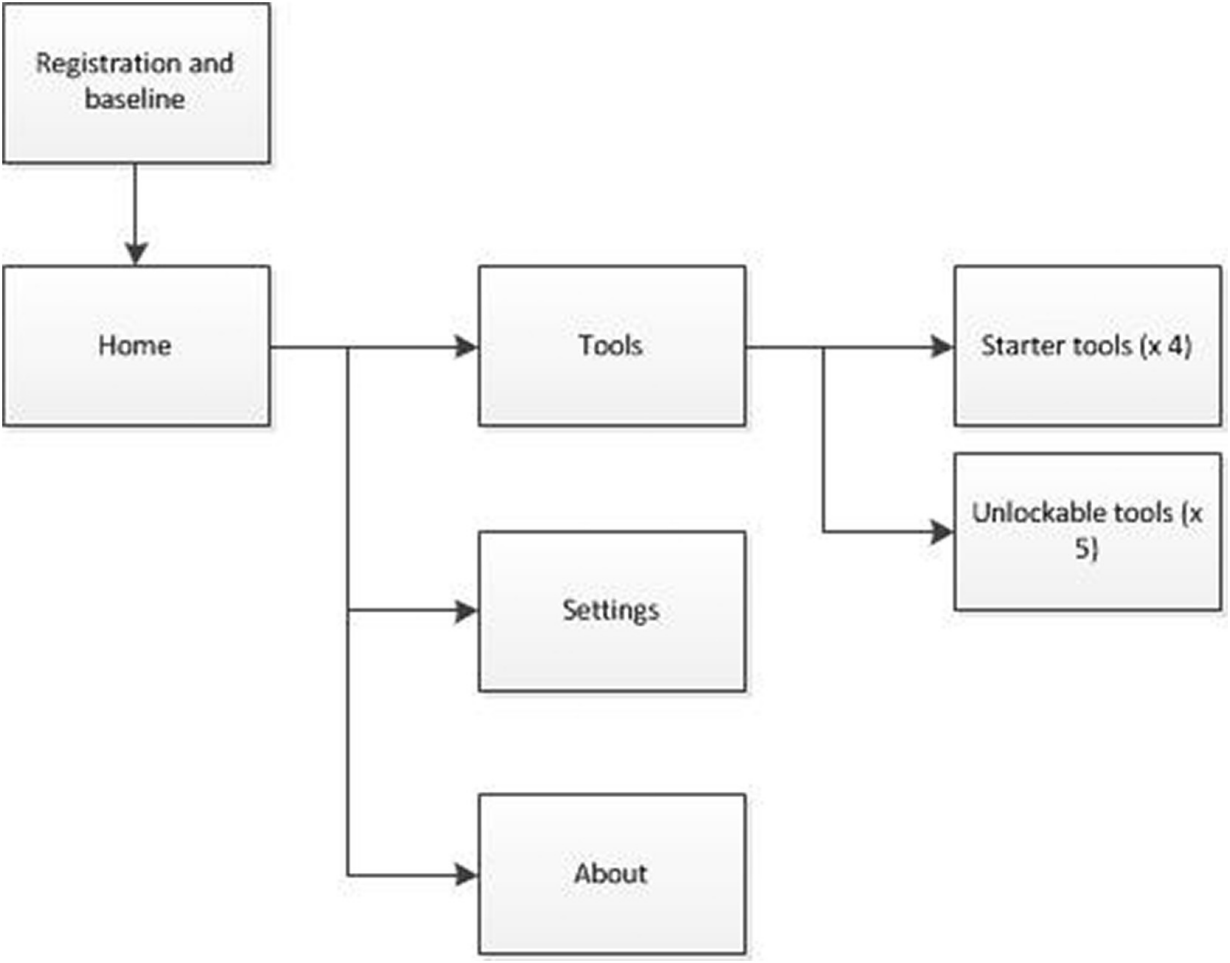
Navigation structure for Healthy Mind (app).

On first access to the Healthy Mind app users were directed to the home screen immediately after completing registration and baseline measures (see Fig. 2). From the home screen users could optionally access an ‘about’ screen that provided details of the aims, contents and benefits of using the app. Four starter tools were initially available on the app (see Table 1). New tools were unlocked each time users completed and rated a tool until all tools were available.

#### 2.3.2 Length and volume of content

The length and volume of textual content was substantially reduced in the app. Specifically, the introduction to the intervention was reduced from 18 screens in the web version to one optional screen in the app version. The longer introduction within the web version was used to normalise the experience of distress and explain the distinction between increasing awareness of thoughts or behaviours versus making changes to thoughts or behaviours. These extended explanations were removed from the app. Similarly, explanations for why and how each tool may be helpful for managing stress were provided as tunnelled content in the web version. These explanations were simplified and made optional within the app. Additionally, the number of tools available was reduced in the app version. The tools provided in the web version comprised those to support increased awareness of thoughts or behaviours and those that supported users to change thoughts or behaviours. Tools that focused on the same types of thought or behaviour were combined within the app (see Table 1).

#### 2.3.3 Push notifications

The interventions were disseminated to participants as part of a wider exploratory trial examining the role of Smartphone-based notifications. Participants using the app were therefore randomised to one of three different versions that delivered notifications at different times with varying frequencies (adaptively based on sensed data, daily between 17.00 and 20.00 and twice weekly between 17.00 and 20.00). Please refer to Morrison et al. (2017) for full details. Participants using Healthy Paths (Web) did not receive email notifications/reminders to log back into the website.

## 3 Materials and methods

### 3.1 Recruitment

Participants were recruited to the study via posters, newsletters and emails circulated within workplaces across the North East, UK. Eligible participants were required to be 18 years of age and have access to the Internet or Android Smartphone. Employers endorsing the study were recruited via UK public health teams and were involved in workplace health activities.

### 3.2 Study design and procedure

Data collection took place between September 2014 and February 2015 and was approved by the University of Southampton ethics committee and research governance office (approval number 12156). All study procedures were fully automated using LifeGuide and LifeGuide Toolbox software (http://www.lifeguideonline.org) and no human contact or support was provided. The Healthy Paths website was accessed via a designated URL. The Healthy Mind app was downloaded to participants' Smartphones via the Google Play Store. Upon initial access to the website and app, participants were presented with an electronic information screen that provided information about the research. Participants were informed that their usage of Healthy Paths or Healthy Mind would be recorded. Participants were also informed that they could delete the app or stop using the website at any time. Informed consent was provided by clicking ‘next’ on this screen. The interventions were designed to be stand-alone. That is, participants could choose to access either the Healthy Paths website and/or the Healthy Mind app.

The automated study procedures were designed to support comparison of usage between the web and app versions of the intervention in a real-world context. Participants were emailed a link to complete an online feedback survey two weeks after their initial registration to the website or app. All participants who completed this survey were entered into a prize draw to win one of four £100 prizes. However, given the design of the study response rates were expected to be low and our primary aim was not therefore to evaluate the effectiveness of the intervention.

### 3.3 Measures

Usage of Healthy Paths and Healthy Mind by each participant was automatically recorded using the LifeGuide intervention authoring software. This included: frequency and duration of logins, frequency and duration of individual page or screen visits, order of individual page or screen visits. Demographic characteristics (age, gender, educational attainment) and email contact were reported by participants at baseline. Self-reported enablement, satisfaction with the intervention, and perceived ease of use were collected via the online feedback survey. Enablement was measured using 3-items adapted from the Patient Enablement Instrument each rated on a 5 point Likert-scale (much better, better, a little better, same, worse): “I am able to cope better with my life”, “I am able to cope better with negative moods”, “I am able to understand my mood better” (Howie et al., 1998). Satisfaction with the intervention was measured using 2-items each rated on 10 point Likert scale (strongly disagree-strongly agree): “The app/website gave me all the advice I needed” and “The app/website was helpful to me”. Perceived ease of use was measured using 2 items adapted from the Technology Acceptance Model – 2 (TAM-2) each rated on a 7 point Likert scale (strongly disagree – strongly agree): “I find the app/website to be easy to use” and “I find it easy to get the app/website to do what I want it to do” (Venkatesh and Davis, 2000).

### 3.4 Analysis

Statistical analyses were performed using IBM SPSS Statistics for Windows version 24.0 (IBM Corp, 2016). Sample characteristics were compared using independent samples *t*-Test (for continuous variables) and Chi-Square (for categorical variables). Usage of the website and app was compared for the two week period following initial registration. This ensured that comparisons were made across the same period of usage and were not biased by participants having used the website or app for varying periods of time based on their initial registration date. Only participants who completed the registration procedure and baseline questionnaire are included in the presented analysis. Usage data were positively skewed and compared using non-parametric tests. Descriptive statistics were computed to summarise the extent of usage of the website and app for the sample as a whole (e.g. median and interquartile range for continuous variables, *n*/% for categorical variables). Specifically, the following variables were computed: duration of use, total number of log-ins, duration of each log-in, and the proportion of participants ceasing use of the intervention within the two week data collection period. Usage variables were compared using Mann-Whitney U (for continuous variables) and Chi-Square (for categorical variables).

Individual participants' flow through the intervention (i.e. order and frequency of page/screen visits) was explored visually using a visualisation tool that is included within the LifeGuide suite of software tools (see Arden-Close et al., 2015). In brief, the visualisation tool generates plots of what intervention content was accessed, in what order, by each individual participant. Data can be exported from the visualisation tool to support statistical analysis of any identified patterns in usage. The aim of the visual exploration was to examine whether there were identifiable differences in patterns of usage between the website and app. Specifically, were there any differences in the extent to which each tool was accessed? Were there any differences in the extent to which different types of tools (i.e. awareness versus behaviour-focused) were accessed?

As expected, response rates to the online feedback survey were low for both web (*n* = 80, 21.00%) and app (*n* = 34, 20.99%) users. Therefore no formal analyses of these data are presented.

## 4 Results

### 4.1 Sample characteristics

In total, 389 participants registered to the Healthy Paths website and 202 participants downloaded the Healthy Mind app. Of these, 381 (97.94%) and 162 (80.20%) participants completed the registration and baseline process on the website and app respectively and were included in the presented analyses. Data on sample characteristics were missing for 20 participants who downloaded the Healthy Mind app; age data for an additional 14 participants was suspected to be false (i.e. <18 and improbable based on reported educational attainment). Table 2 provides a summary of the sample characteristics. The age range of web users was 20 to 69 years compared to 18 to 62 years for app users. The average age of web users was significantly higher than app users, *t*(507) = −7.74, *p* < 0.001. A significantly higher proportion of web users were female (around three quarters) compared to app users (just over half), *χ*^2^(1, *n* = 523) = 23.71, *p* < 0.001. Around half the users of both the website (*n* = 207, 54.43%) and the app (*n* = 71, 50.00%) were educated to degree level or higher. The proportions of web and app users educated to degree level or higher did not differ significantly, *χ*^2^(1, *n* = 523) = 0.78, *p* = 0.38.

**Table 2:** Sample characteristics.

	Healthy Paths (*N* = 381)	Healthy Mind (*N* = 142)^a^
Age (years): *M* (*SD*)	44.75 (10.60)	36.38 (10.53)^b^
Gender (female): *n* (%)	305 (80.05)	84 (59.15)
Educational attainment		
No formal qualifications	5 (1.31)	14 (9.86)
GCSE	51 (13.39)	26 (18.31)
A-level	52 (13.65)	16 (11.27)
Diploma, vocational or professional qualification	54 (14.17)	14 (9.86)
Undergraduate degree	121 (31.76)	46 (32.39)
Postgraduate degree	86 (22.57)	25 (17.61)
Other	12 (3.15)	1 (0.70)

a Baseline data were missing for n = 20 participants.

b Based on *n* = 128 as age data suspected to be false for *n* = 14.

### 4.2 Summary-level usage patterns

Table 3 compares the core usage variables for the website and app. Web users spent significantly longer on the intervention compared to app users, *U* = 19,740.00, *p* ≤ 0.001. Web users logged in to the intervention significantly fewer times than app users, *U* = 13,059.50, *p* ≤ 0.001, but spent significantly longer on the intervention at each login, *U* = 7731.00, *p* < 0.001. A significantly higher proportion of web users ceased use of the intervention within 2 weeks compared to app users, *χ*^2^(1, *n* = 543) = 36.90, *p* < 0.001.

**Table 3:** Summary-level usage of Healthy Paths website and Healthy Mind app.

Usage variable	Healthy Paths (*N* = 381)	Healthy Mind (*N* = 162)
Duration of use, mins (*Mdn*, *IQR*)	10.52 (13.53)	5.23 (8.69)
Number of log-ins (*Mdn*, *IQR*)	1.00 (1.00)	2.00 (2.00)
Duration of each log-in, min (*Mdn*, *IQR*)	8.56 (9.76)	2.12 (2.58)
Ceased use within 2 weeks (*n*, %)	313 (82.15%)	69 (42.59%)

### 4.3 Tool usage

**Fig. 3:**
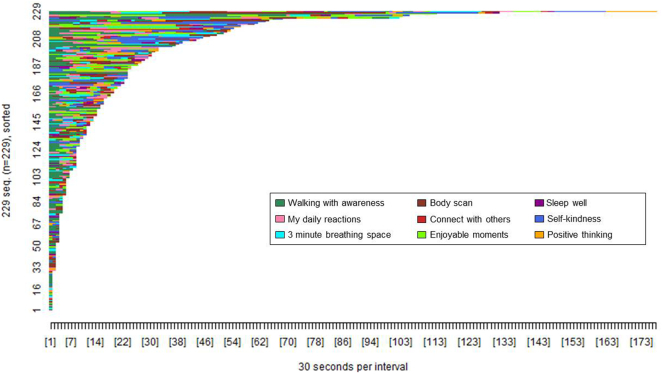
Pattern of tool access by Healthy Paths (web) users (*n* = 229). The x-axis shows the passage of time in 30 s intervals. The y-axis shows each participant who accessed tools on the website. Each colour shows access to a specific tool as per the figure legend.

**Fig. 4:**
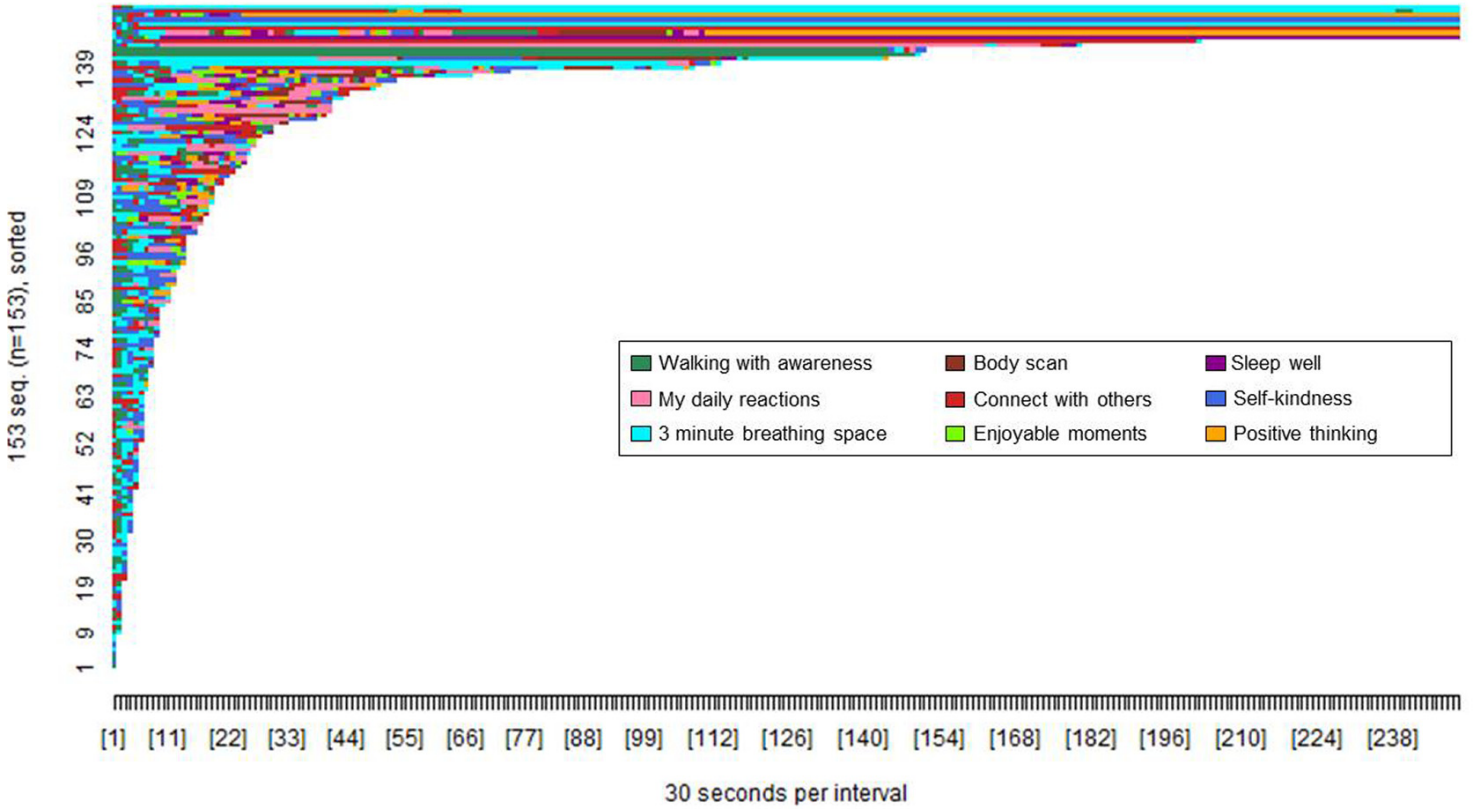
Pattern of tool access by Healthy Mind (app) users (*n* = 153). The x-axis shows the passage of time in 30 s intervals. The y-axis shows each participant who accessed tools on the app. Each colour shows access to a specific tool as per the figure legend.

Fig. 3, Fig. 4 show the pattern of tool access by web and app users within the 2 week period post-registration. A greater proportion of app users accessed at least one of the available tools (*n* = 153, 94.44%) compared to web users (*n* = 229, 60.10%), *χ*^2^(1, *n* = 543) = 64.26, *p* < 0.001.

The difference in colour tones between Fig. 3, Fig. 4 show that the proportion of participants accessing each tool type varied between the website and app. The designated starter tools on the app (3 min breathing space, walking with awareness, self-kindness, and connect with others) were each accessed by 70 to 80% of app users compared to the non-starter tools, each accessed by <30% of app users (see Table 4). In contrast, each tool on the website was accessed by 20 to 40% of web users, with the greatest proportion of users accessing the walking with awareness tool. A significantly greater proportion of app users accessed all four of the designated starter tools (*n* = 68, 44.44%) compared to web users access of those same tools (*n* = 17, 7.42%), *χ*^2^(1, *n* = 382) = 72.66, *p* < 0.001.

**Table 4:** Proportion of participants (*n*, %) accessing each tool.

	Healthy Paths (*n* = 229)	Healthy Mind (*n* = 153)
Walking with awareness^a^	142 (62.01)	117 (76.47)
My daily reactions	98 (42.79)	43 (28.10)
3 minute breathing space^a^	72 (31.44)	126 (82.35)
Body scan	76 (33.19)	30 (19.61)
Connect with others^a^	53 (23.14)	107 (69.93)
Enjoyable moments	82 (35.81)	32 (20.92)
Sleep well	48 (20.96)	29 (18.95)
Self-kindness^a^	75 (32.75)	111 (72.55)
Positive thinking	55 (24.02)	36 (23.53)

a Starter tools in the app.

**Fig. 5:**
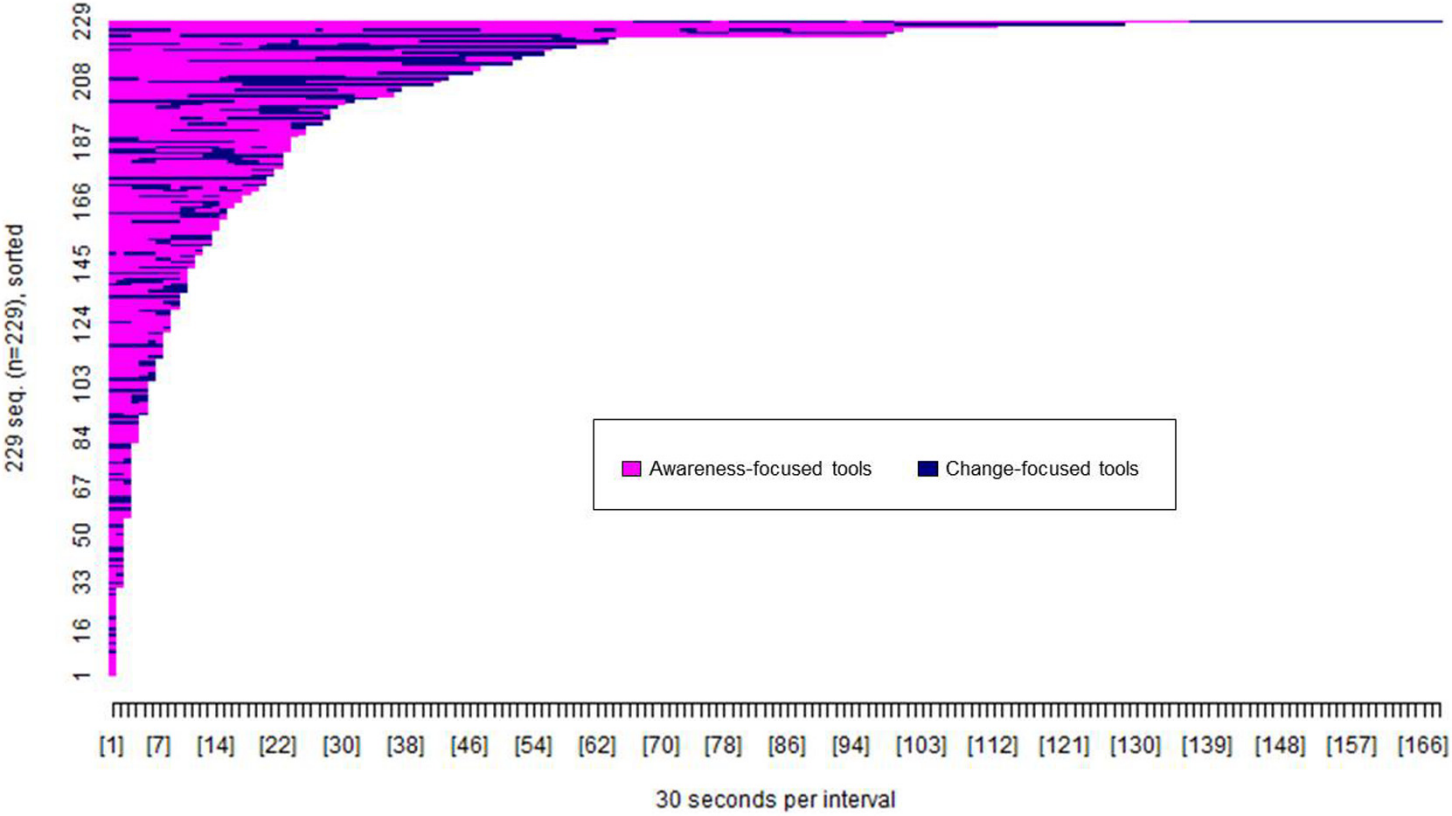
Pattern of access to awareness and change-focused tools by Healthy Paths (web) users (*n* = 229). The x-axis shows the passage of time in 30 s intervals. The y-axis shows each participant who accessed tools on the website. Each colour shows access to a specific tool type as per the figure legend.

**Fig. 6:**
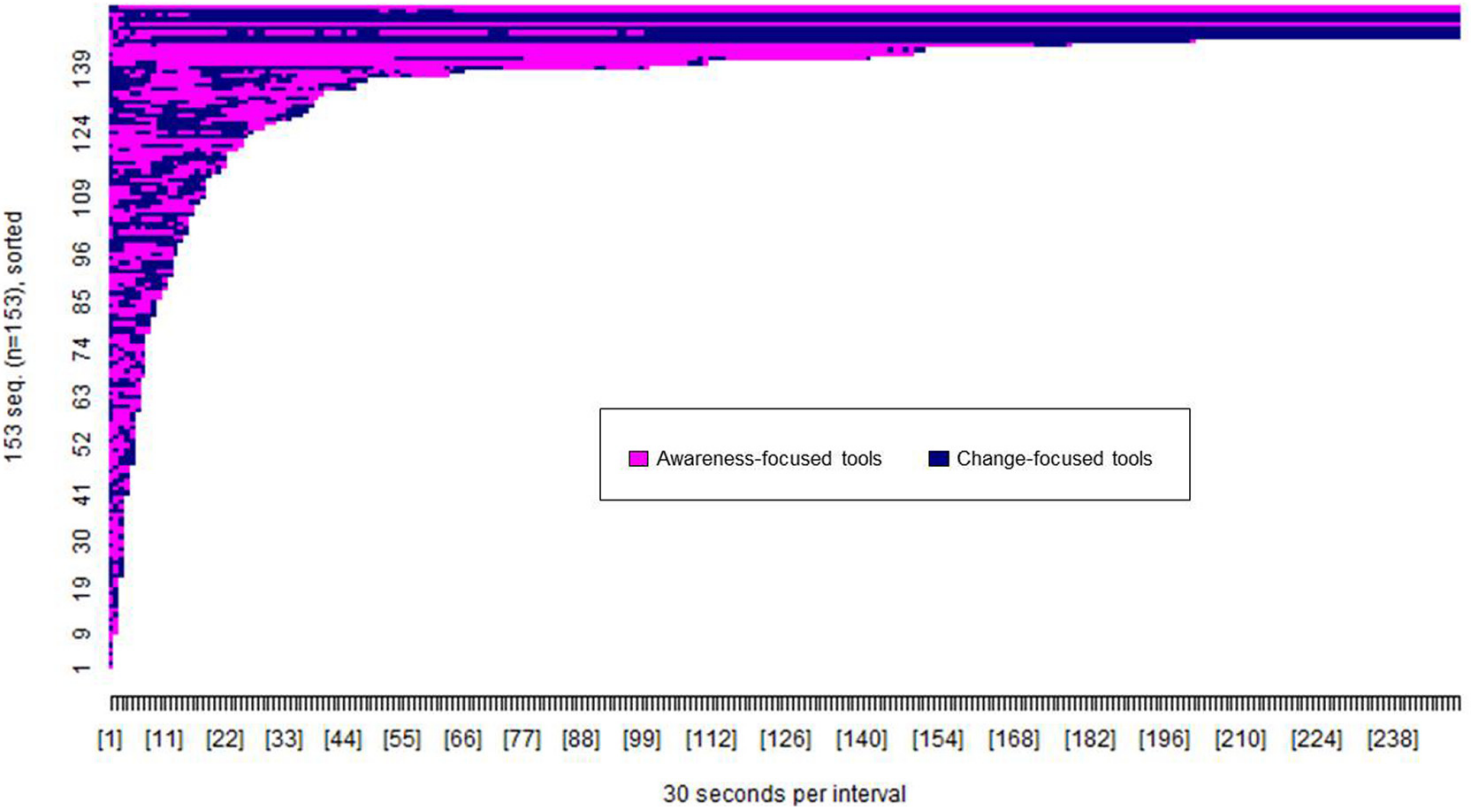
Pattern of access to awareness and change-focused tools by Healthy Mind (app) users (*n* = 153). The x-axis shows the passage of time in 30 s intervals. The y-axis shows each participant who accessed tools on the app. Each colour shows access to a specific tool type as per the figure legend.

Fig. 5, Fig. 6 show the pattern of access to each tool type (awareness versus change focused) by web and app users. The difference in colour tone between Fig. 5, Fig. 6 again illustrate that the pattern of tool access varied between the website and app. A greater proportion of app users accessed both types of tool (*n* = 126, 82.35%) compared to web users (*n* = 92, 40.17%), *χ*^2^(1, *n* = 382) = 66.60, *p* < 0.001, whereas a greater proportion of web users accessed only awareness-focused tools (*n* = 108, 47.16%) compared to app users (*n* = 16, 10.46%), *χ*^2^(1, *n* = 382) = 56.36, *p* < 0.001. There were no differences in the proportion of app users (*n* = 11, 7.19%) and web users (*n* = 29, 12.66%) accessing only change-focused tools, *χ*^2^(1, *n* = 382) = 2.93, *p* < 0.09.

## 5 Discussion

Usage of a web-based stress management intervention differed from usage of an app-based intervention that shared the same underlying theoretical components. Web users logged in fewer times for a longer period of time whereas app users logged in more frequently for shorter periods of time. This usage pattern fits with insights from prior qualitative and mixed-methods work suggesting that apps may be used more sporadically (Dennison et al., 2013; Morrison et al., 2014).

However, there were important differences in what elements of the intervention were accessed. Nearly all app users accessed at least one of the tools compared to just over half the web users. App users chose to access a higher number of tools from a narrower range (e.g. designated starter tools) whereas web users chose to access a fewer number of tools from a wider range. These findings suggest that the deliberate design choices (e.g. enabling quicker access to tools, restricting initial choice of tools, prompting tool use) may have influenced subsequent usage patterns. This adds to an emerging literature illustrating that methods of digital intervention delivery are associated with differences in uptake, usage and experience of the intervention (Quinonez et al., 2016).

Recent conceptual models argue that usage of and engagement with digital behaviour change interventions is directly influenced by both the design of the intervention and the study context, including user characteristics (Perski et al., 2016). The usage patterns observed in this study appear to fit with this model; enabling easier and quicker access to the core intervention content (i.e. tools), the introduction of a game-based element (tool unlocking) and/or prompting tool use through notifications may have encouraged app users to access more content in a shorter time. Additionally, the difference in sample characteristics between web and app users may have also contributed to the differences in the observed usage patterns. Data from this observational study cannot test causal relationships between design factors, user characteristics and usage. Nonetheless, these findings do illustrate that decisions about the delivery platform for health behaviour interventions are not trivial and should be empirically and theoretically informed, taking into account the lifestyles and characteristics of the intended target population.

This study employed two different approaches to analysing intervention usage that each provided useful and complementary data. Summative usage metrics (e.g. total time spent on the intervention, number of logins etc.) were useful for determining the overall amount or frequency with which each version of the intervention was used. However, these broad metrics could not provide more detailed insights about the extent to which the core intervention content was accessed over time. Visual exploration of individual-level usage patterns enabled more efficient detection of key differences in patterns of usage that could then be examined statistically. This study adds to a growing literature emphasising the benefits of moving beyond summative usage metrics (Arden-Close et al., 2015; Morrison and Doherty, 2014). Relying on broad summative usage metrics may mask important differences in how digital interventions are engaged with over time and the factors associated with different levels of engagement. Future empirical work may benefit from routinely and systematically combining multiple methods of analysing usage that will guide more informed decisions about optimal intervention design and delivery. A cumulative science around digital intervention usage is also constrained by the lack of comparability across different studies (Nelson et al., 2016). The development of guidance to support systematic and rigorous analysis of usage will help to ensure that reported analyses are comparable across studies and potentially generate broader insights or recommendations that are applicable across interventions, behaviours or contexts.

There is an underlying assumption in many usage analyses, including the analyses presented in this paper, that more or greater usage is optimal. The relationship between usage and health-related outcomes is complex. Although there is evidence that greater exposure to intervention content can be associated with intended intervention outcomes (e.g. Van Gemert-Pijnen et al., 2014), this is not true in all cases (e.g. Saul et al., 2016). There is now growing consensus that we need to identify and promote an effective level of engagement with digital interventions (Yardley et al., 2016). That is, a level of engagement that is sufficient for supporting the user to achieve the desired outcomes. For example, in this study, it is possible that web users accessed a smaller number of tools because they received sufficient support from the extended introductory pages or were better able to identify which tool would or would not help them. Alternatively, app users may have accessed more tools quickly that they could then apply offline, as needed. What constitutes “effective engagement” may vary for different user groups, behaviours or intervention settings. Looking forwards, where possible, analyses of usage should seek to identify these thresholds for effective engagement (e.g. Ainsworth et al., 2016).

This was an observational study. Participants were not randomised to receive the web or app versions of the intervention and user characteristics differed significantly between the two samples. Additionally, the website and app were not directly comparable and a number of design modifications were introduced. This study has generated important research questions, but it is not possible to draw definitive conclusions about how specific design choices influenced intervention usage. Further empirical work is needed to replicate the differences observed in usage between different digital platforms (e.g. web versus app) and definitively test the explanations proposed for the differences (e.g. design features versus user characteristics). The analyses were exploratory and illustrate one possible approach to interpreting the data. As expected, response rate to the online follow-up survey was low. Therefore it was not the aim of this study to examine the relationship between intervention usage, satisfaction with the intervention and health-related outcomes. Thus no conclusions are drawn about effective levels of usage.

## 6 Conclusions

Findings from this study suggest that changing the way in which intervention content is delivered (e.g. when adapting an intervention for delivery via web and app) may lead to important differences in how the underlying theoretical content is used and received. Users of a stress management app accessed more of the core intervention content in a shorter time compared to users of a website. Additional research is needed to test the extent to which variations in usage are influenced by differences in intervention design and delivery between the two platforms or differences in user characteristics. Combining broad summative usage metrics (e.g. total time spent on the intervention, number of log-ins) with more detailed individual-level data on how specific parts of the intervention are used over time can provide a more informed interpretation of intervention usage and engagement.

## Conflict of interests

None declared.
